# *Delonix regia* pollen extract enhances growth and bioactive compound production in *Coriandrum sativum* by upregulating key biosynthetic genes

**DOI:** 10.1038/s41598-025-33993-6

**Published:** 2026-01-19

**Authors:** Eman M. Bassiouni, Dalia Y. El-Berawey, Salwa M. Abdel Rahman, Eman M. M. Eldebawy

**Affiliations:** 1 Department of Botany and Microbiology, Faculty of Science, Alexandria University, Alexandria, Egypt; 2 Biology and Geology Department, Faculty of Education, Alexandria University, Alexandria, Egypt; 3 Botany and Microbiology Department, Faculty of Science, Damanhour University, Damanhour, Egypt

**Keywords:** Biostimulant, Coriander, Gene expression, Pollen grains, Royal poinciana, Sustainable agriculture, Biochemistry, Biotechnology, Physiology, Plant sciences

## Abstract

The application of biostimulants represents a sustainable strategy to enhance crop productivity and resilience. This study investigated the efficacy of *Delonix regia* pollen aqueous extract as a biostimulant on coriander (*Coriandrum sativum*). Seeds were primed with different concentrations of the extract (0%, 0.5%, 1%, and 1.5%) for 24–48 h. The 1% extract applied for 48 h was the most effective treatment, significantly increasing shoot fresh and dry weight, shoot length, and the content of total protein, phenolics, flavonoids, and terpenes. This treatment also led to a significant upregulation of the key biosynthetic genes Chalcone synthase (CHS) and geranylgeranyl pyrophosphate synthase (GGPS) by 1.4-fold and 2.1-fold, respectively. Principal component analysis confirmed a positive correlation among shoot fresh weight, total protein, terpene content, and GGPS expression. These findings demonstrate that *D. regia* pollen extract is a potent biostimulant that enhances coriander growth and the production of valuable bioactive compounds through the modulation of key metabolic pathways.

## Introduction

 The use of biostimulants in sustainable agriculture is growing exponentially, offering a valuable approach to enhance plant development, optimize nutrient uptake, improve stress tolerance, and increase the production of plant active constituents. As the demand for sustainable agricultural practices grows, the use of biostimulants is increasing exponentially to enhance plant development and reduce environmental impact^1–4^. Recent comprehensive reviews, such as that by Matthews et al.^5^, highlight the diverse mechanisms and significant potential of biostimulants for improving crop performance, underscoring the need for exploring novel natural sources. Advanced analytical techniques have revealed the presence of anthocyanins and other polyphenolic compounds in the flower extracts, which exhibit significant antioxidant activity. These compounds can effectively neutralize free radicals, potentially offering protection against diseases associated with oxidative stress^6,7^.

The application of pollen grains as a biostimulant can be considered an effective strategy to enhance crop yield and improve nutritional value while reducing the environmental impact of agricultural practices^8^. Abou-Sreea and Yassen^9^ demonstrated that application of pollen as a biostimulant can enhance plant growth parameters, including height, stem diameter, leaf dimensions, and biomass accumulation, ultimately improving productivity of *Strelitziareginae* Ait. plants. Moreover, Taha et al.^10^ reported that utilization of pollen extracts improved plant growth and water use efficiency, elevated antioxidant enzyme activities, and promoted the production of essential oil in *Ocimum basilicum* under drought stress. Furthermore, pollen extracts can enhance flowering by regulating flowering time, increasing inflorescence numbers, and improving flower and fruit development, and seed production, which in turn improve plant production^9^.

The genus *Delonix* Raf. belongs to family Fabaceae, subfamily Caesalpinioideae. The Fabaceae family is an important family of flowering plants with medicinal and economic values^11^. The legume family is widely distributed in temperate and tropical climates^12^. The genus *Delonix* comprises approximately 12 accepted species, 9 of them are endemic to Madagascar. The native range of this genus is Egypt to Tanzania and West India, Madagascar^13^. Regarding chemical composition, different types of terpenes, flavonoids, cyanogenic glycosides, and phenylpropanoids have been reported in family Fabaceae^14,15^. Silva et al.^16^ conducted an extensive analysis of flavonoid diversity across Fabaceae, emphasizing their presence in diverse taxa.d.

*Delonix regia* (Bojer ex Hook.) Raf., syn. *Poinciana regia* Bojer ex Hook., is a deciduous tree (about 10 m) with compound bipinnate leaves, crimson to orange flowers, and fruit legume. This plant is cultivated for shade and ornamental purposes, as well as for its great ethnomedicinal value It is native to Madagascar^17^. The Royal Poinciana is considered one of the five most beautiful flowering trees throughout the world^18^; it supports biodiversity and soil stabilization. The different parts of *D. regia* uniquely contribute to its therapeutic potential, exhibiting antidiarrheal, hepatoprotective, antioxidant, anti-inflammatory, and antimicrobial efficiency^19^. Therefore, it has been applied in folk medicine in many civilizations as a treatment for constipation, inflammation, arthritis and rheumatism. Flowers of *D. regia* have been used as traditional herbal remedies for gynecological disorders and they are also used as tablet binders^18^. Based on phytochemical studies, many compounds have been detected in different parts of this plant species, such as flavonoids, alkaloids, phenolics, anthocyanins, fatty acids, tannins, sterols, and triterpenes^19–23^. Moreover, protein and amino acids have been also reported^24^.

*Coriandrum sativum* L. (Coriander), is a member of the Apiaceae family, it is an annual herb native to the Mediterranean and Middle East, now found in various regions^25–27^. It’s rich in bioactive compounds like polyphenols, terpenes, and fatty acids, making it a healthy source of nutrition, and medicinal uses^28–31^. *C. sativum* has various health benefits, including antioxidant, antimicrobial^32^, and anti-inflammatory properties^33^. It supports heart health, digestion, and blood sugar management^34,35^. Coriander may also improve memory, relieve digestive issues, and help prevent obesity, metabolic syndrome, and diabetes^28,34^. It is used as a flavoring agent to enhance the taste of various culinary preparations^36^. Its bioactive compounds also make it a potential natural preservative to extend shelf life and prevent foodborne illness^37^. Coriander can be used in various forms, including fresh or dried leaves, whole seeds, or ground seeds^38^.

Despite the significant increase in the utilization of biostimulant worldwide, research on the use of pollen as a plant biostimulant is emerging, and more studies are needed to fully understand its potential benefits on crop yield and food security. In addition, most studied focus on foliar application of pollen grain extract rather than seed priming^3,8^. Therefore, the current study aimed to explore the physiological, biochemical and molecular responses of *Coriandrum sativum* to *Delonix regia* pollen extract. To the best of our knowledge, this study is the first to investigate the efficacy of *D. regia* pollen aqueous extract a seed primer on the growth and the production of valuable bioactive compounds in *C. sativum.*

## Materials and methods

### Collection of *Delonix regia* pollen grains

The flowers of *D. regia* were gathered from the garden of Faculty of Science, Alexandria University, Egypt in July 2024. All dehisced anthers of open flowers or fully mature buds were separated, then dried and frozen.

### Photographing and measurements of pollen grains

The pollen grains of *D. regia* were investigated under light microscope (OPTICA, B-150D) designed with internal USB digital-Video Camera. Furthermore, the pollen grains were examined under JEOL-JSM.I T200 Series scanning electron microscope (SEM) by adding them on a stub coated with 30 nm Gold in a Polaron JFC-1100 coating unit. The examination under SEM was performed in the electron microscope unit, Faculty of Science, Alexandria University, Egypt.

### Determination of total phenolic content of *Delonix regia* pollen grains

The total phenolic content of the aqueous methanol extract of *D. regia* pollen grains was determind following the Folin-Ciocalteu’s method^39^. The reduction of the Folin-Ciocalteu by phenolic compounds under alkaline conditions resulted in a blue color, which was detected at 760 nm in a spectrophotometer (Shimadzu UV-VIS2040 PC, Tokyo, Japan). The results were expressed as milligrams of gallic acid equivalent (GAE) g^− 1^ dry mass^40^.

### Determination of total flavonoids content of *Delonix regia* pollen grains

The total flavonoid content was determined using the colorimetric technique following Adusei et al.^41^. Pollen extract was mixed with distilled water, sodium nitrite, aluminum chloride, and sodium hydroxide. Absorbance was measured at 510 nm using a spectrophotometer. The total flavonoid content was calculated as mg quercetin equivalents per gram of sample.

### Determination of total antioxidant capacity (TAC) of *Delonix regia* pollen grains

The total antioxidant capacity of pollen extract was measured spectrophotometrically at 695 nm by the phosphomolybdenum assay as mentioned by Anokwah et al.^42^. Ascorbic acid was used as a positive reference standard. The average values were calculated from three trials for each concentration. The antioxidant capacity was estimated using the following formula:

$$The{\text{ }}antioxidant{\text{ }}capacity{\text{ }}\% {\text{ }}={\text{ }}A{\text{ }}sample{\text{ }} - {\text{ }}A{\text{ }}control{\text{ }}/{\text{ }}A{\text{ }}sample{\text{ }} \times 100$$

Where, A sample is the absorbance of the sample and *A control* is the absorbance of the control.

### Determination of DPPH scavenging assay of *Delonix regia* pollen grains

The antioxidant activity of pollen extract was determined using the DPPH free radical scavenging assay described by Krzyczkowska and Kozłowska^43^. Different concentrations of pollen extract were mixed with methanol solution and DPPH radicals. The reaction mixtures were incubated in the dark for 30 min at room temperature, and their absorbance was measured at 517 nm using a UV–VIS spectrophotometer. The blank sample included methanol instead of extract.

% Radical scavenging activity was calculated by using the following formula:

$$\% {\text{ }}Inhibition\,=\,A{\text{ }}Control{\text{ }} - {\text{ }}A{\text{ }}Test{\text{ }}/{\text{ }}A{\text{ }}Control \times 100$$

Where *A control*: absorbance of control; *A Test*: absorbance of samples.

### Experimental design and treatments

#### Pollen extract Preparation

The aqueous pollen extract was prepared by soaking the finely powdered crushed anthers of *Delonix regia* in distilled water. Three different concentrations were prepared, specifically 0.5, 1, and 1.5% (w/v).

#### Seed priming

Coriander seeds (*Coriandrum sativum*) were obtained from the local market. Before germination, the seeds were immersed in a 0.1% sodium hypochlorite solution for 3 min before being thoroughly washed with distilled water. The sterilized seeds were then immersed in the aqueous pollen extract with different concentrations for 24–48 h. The experimental design involved the following treatments: C = no pollen treatment, T1 = seed priming in 0.5% pollen solution, T2 = seed priming in 1% pollen solution, T3 = seed priming in 1.5% pollen solution. The experiment was conducted in the greenhouse of the Faculty of Science, Damanhour University, using a completely randomized design. The pots were incubated in normal environmental conditions (photoperiod of 12 h light/12 h dark, 28/23 ± 2 C light/dark temperature; light intensity PPFD, 23 µmol m^− 2^s^− 1^). Three replicates from the leaves of each treatment were taken for physiological, biochemical and molecular analyses.

### Determination of total carbohydrates and protein content of *Coriandrum sativum*

Total carbohydrates and total protein were determined as described by methods of Dubois et al.^44^ and Hartree^45^, respectively.

### Determination of total phenolic content of *Coriandrum sativum*

The total phenolic content of *C. sativum* was estimated as described by^40^.

### Determination of total flavonoids content of *Coriandrum sativum*

The total flavonoids content in the methanol extracts of *C. sativum* was quantified using a spectrophotometric method, as described by Quettier et al.^46^. The flavonoid content in extracts was determined and expressed in terms of quercetin equivalent (µg quercetin/g dm^− 1^(.

### Determination of total terpenes content of *Coriandrum sativum*

Terpenoids were extracted from a dry plant sample using methanol, then a chemical reaction with chloroform and sulfuric acid was performed to produce a reddish-brown color indicative of their presence. The resulting solution’s absorbance was measured at 538 nm. Finally, the total terpenoids content was quantified using a standard curve of Linalool in methanol, and the results were expressed in mg g⁻¹ dm^47^.

### Expression analysis of flavonoids and terpenoids main genes in *Coriandrum sativum* under treatment with *Delonix regia* pollen extract

#### Synthesis of cDNA and RNA extraction

TRIZOL reagent was prepared as it cited in (Rodríguez-Ezpeleta et al. 2009) was used to manually extract total RNA^48^. The concentration and purity of RNA were measured at 260 and 280 nm using Nano Drop 2000/2000c Spectrophotometer (Thermo Scientific, USA). To conduct reverse transcription procedures, the COSMO cDNA synthesis Kit (willowfort) was utilized. One microliter of total RNA (300ng), four microliters of cDNA Reaction Buffer (5x), and one microliter of RT Enzyme Mix were included in each 20 µL reverse-transcription mixture. To inactivate the reverse transcriptase, the samples were incubated for five minutes at 25 °C, fifteen minutes at 55 °C, and five minutes at 85 °C (http://willowfort.co.uk)^49^.

#### Real-time PCR primers and reaction

Chalcone synthase (CHS) and geranylgeranyl pyrophosphate synthase (GGPS) were the target genes in *C. sativum* treated with *D. regia* pollen extract. The expression of these genes was established by normalizing with the control gene, glyceraldehyde-3-phosphate dehydrogenase (GAPDH), using specific primers for amplification (Table 1). The primers for GGPS were designed in accordance with GGPS genes (MG762024.1, DQ192184.1, OM732401.1) by employing the Jalview program^50^ and proved by BLAST (https://www.ncbi.nlm.nih.gov/tools/primer-blast/index.cgi). However, CHS and GAPDH gene primers were extracted from previous studies^51,52^. All primers were synthetized by WillowFort Company (United Kingdom). Quantitative RT-PCR (Applied Biosystems StepOne™ instrument) using the HERA SYBR^®^ Green qPCR master mix (http://willowfort.co.uk) to determine the expression of the CHS and GGPS genes. 10 µL of qPCR Kit Master Mix, 1 µL of 10 pmol primer (F), 1 µL of 10 pmol primer (R), 1 µL of cDNA and 7 µL H_2_O_2_ made up the reaction mixture, which had a total volume of 20 µL. The following methodology was applied to the PCR reactions: 95 °C for 2 min (one cycle). 95 °C for 10 s, 60 °C for 30 s (50 cycles), and transcript levels were computed by normalizing the expression level of main genes using the in-run GAPDH gene as a control. With ΔCt = Ct (main gene) - Ct (control gene), where relative transcription level = 2^−ΔCt^^53^. Relative gene expression was calculated using the 2 − ΔCt. method, assuming comparable amplification efficiencies based on primer design parameters and melting curve.

**Table 1:** Sequences of primers of target and reference genes used in RT-PCR.

Gene	Primer sequence	
**CHS**	**F**	5-ACTGGAACTCCTTCTTCTGGA-3
	**R**	5-AACCCGAAAAGAACACCCCA-3
**GGPS**	**F**	5-CATGCACGACGATCTCCCTT-3
	**R**	5-ACAAGGTGGAAAGCTAGGGC-3
**GAPDH**	**F**	5-TCCTACGATGCCATCAAGGC-3
	**R**	5-ACGAAATCGGTGGAGACGAC-3

### Data analysis

An analysis of data variance was performed utilizing a completely randomized design with three replicants. The comparison of average values was executed based on the Least Significant Difference (LSD) test at a significance level of *p* < 0.05 according to Sokal and Rohlf’s technique^54^, employing SPSS software. Graphical representations were created using Excel. Furthermore, principal component analysis (PCA), and correlation were generated using R software v.4.3.1^55^.

## Results

### Morphological characteristics of *Delonix regia* pollen grains

**Fig. 1:**
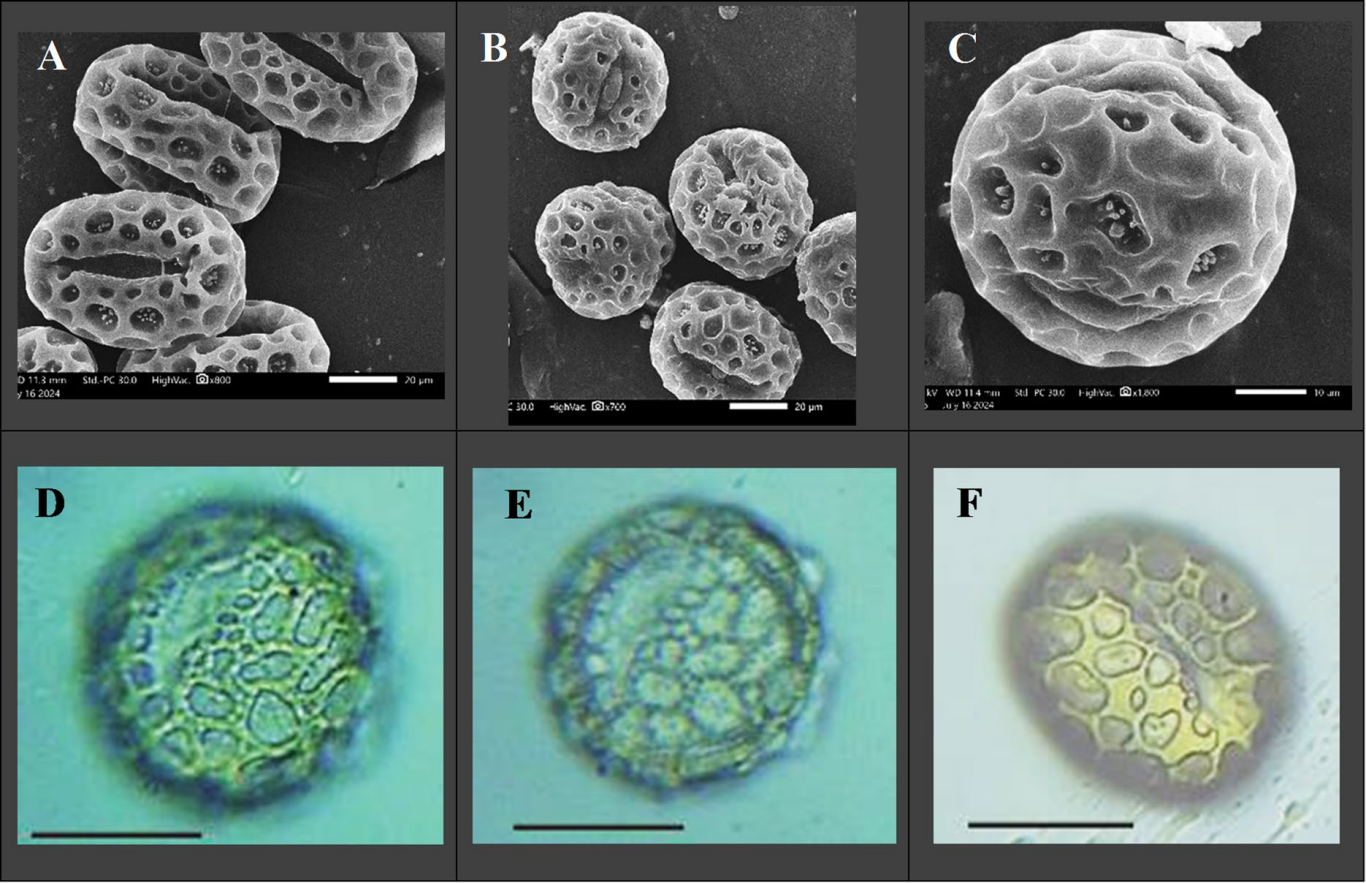
Pollen grains of *Delonix regia*: **A-C**. Pollen grains photographed by SEM; (A) Equatorial view, (B) Equatorial and polar views, (C) Baculate, reticulate exine, **D-F**. Pollen grains in equatorial view taken by light microscope (scale bar = 20 μm).

It is obvious that the flowers of *Delonix regia* shed pollen as monads. The pollen grain is isopolar and radiosymmetric. Measurements have been taken for an average of 20–30 readings. For dry pollen, the polar axis ranges from 60.00 to 70.31 μm (65.59 ± 2.65), whereas the equatorial diameter ranges from 40.00 to 50.00 μm (43.64 ± 2.32). P/E ratio varies from 1.31 to 1.61 (1.50 ± 0.08). Therefore, the shape of pollen is almost subprolate to prolate. Amb is convex triangular. Pollen size in accordance with Erdtman^56^ is large (50–100 μm). The aperture type is tricoplorate. The colpus length ranges from 36.36 to 56.25 μm (43.99 ± 5.36). Exine shows reticulate pattern under light microscope, while SEM revealed detailed baculate, reticulate sculpture. Lumina is variable in shape and reduced in size along the colpi margins (Fig. 1).

### Total phenolic and flavonoid contents, and antioxidant activity of *Delonix regia* pollen grains

The total phenolic, flavonoid contents, and antioxidant activity of the methanolic extract of *Delonix regia* pollen grains were evaluated; the results are presented in Table 2. The total phenolic and flavonoid contents in *D. regia* pollen extract were determined to be 1.826 and 0.636 mg quercetin/g. dm, respectively. The results demonstrated that *D. regia* pollen extract possessed potent free radical scavenging activity and high total antioxidant capacity at all concentrations tested (100, 200, and 250 mg/mL).

**Table 2:** Total phenolic and flavonoid contents, and antioxidant activity of *Delonix regia* pollen grains.

Total phenolic content 1.826 mg GAE/g. dm		
Total flavonoid content 0.636 mg quercetin/g. dm		
**Total antioxidant capacity (TAC)**		
100 (mg/mL)	200 (mg/mL)	250 (mg/mL)
90.85%	89.27%	86.04%
**DPPH scavenging activity**		
100 (mg/mL)	200 (mg/mL)	250 (mg/mL)
86.55%	86.42%	82.41%

GAE = gallic acid equivalent; dm = dry matter.

### Effect of seed priming in *Delonix regia* pollen aqueous extract on growth parameters of *Coriandrum sativum*

**Fig. 2:**
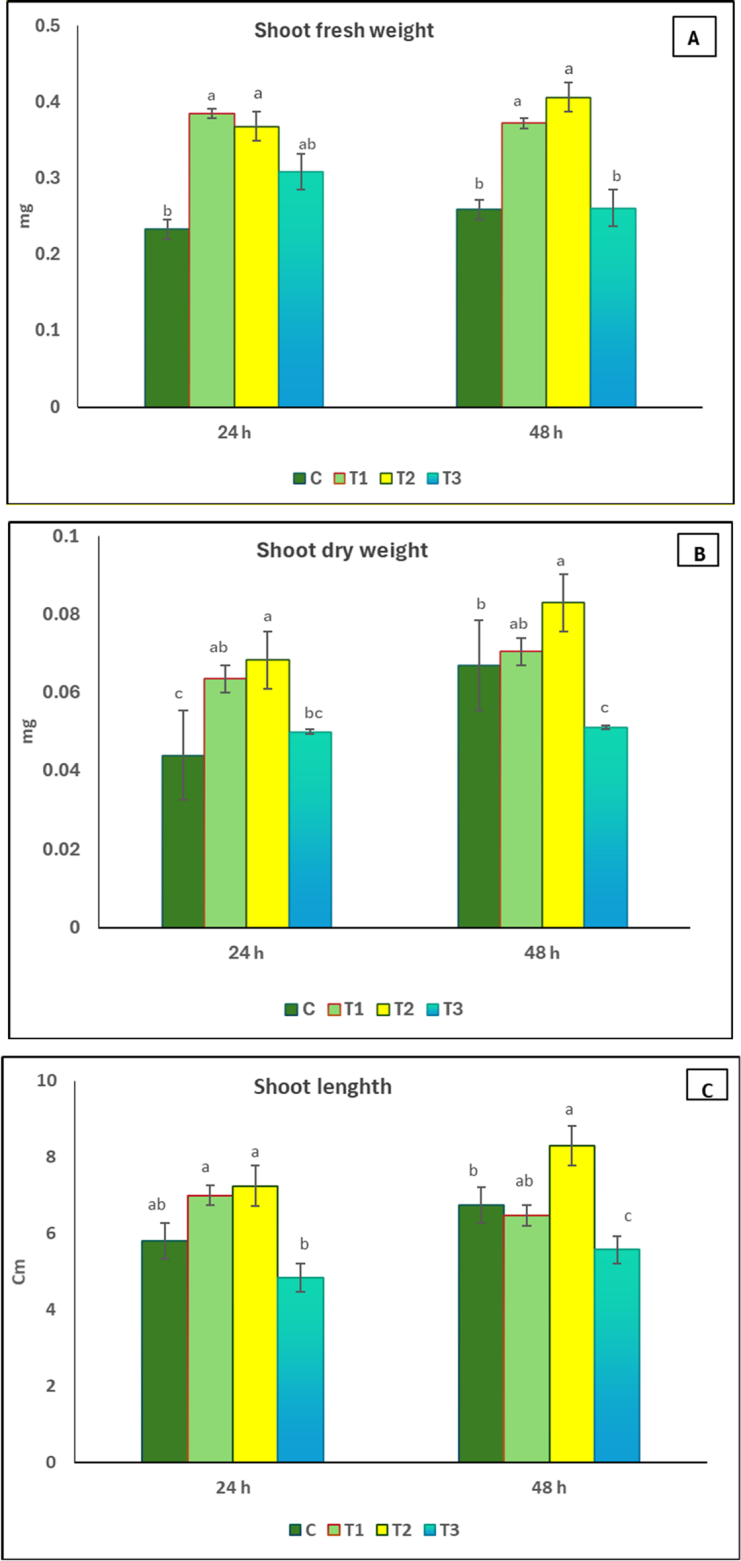
Effect of seed priming in *D. regia* pollen aqueous extract on shoot fresh weight (**A**), shoot dry weight (**B**), and shoot length (**C**) of *C. sativum*. C = no pollen treatment; T1, T2 and T3 = seed priming in 0.5, 1 and 1.5% pollen solution, respectively. The values reported in the figure are means ± standard deviation. Different letters on the bars are significantly different as evaluated by LSD Test for each pollen extract time (24 and 48 h) separately.

The effect of application of *D. regia* pollen extract on shoot fresh, dry weight, and length of *C. sativum* is presented in Fig. 2. Generally, the results indicated that a 48-hour seed priming period in *D. regia* pollen aqueous extract was more effective than a 24-hour period in enhancing growth parameters. Regarding pollen extract concentration, 1% (T2) was the most effective concentration, while 1.5% (T3) was the least effective one.

### Effect of seed priming in *Delonix regia* pollen aqueous extract on total carbohydrate and total protein contents of *Coriandrum sativum*

**Fig. 3:**
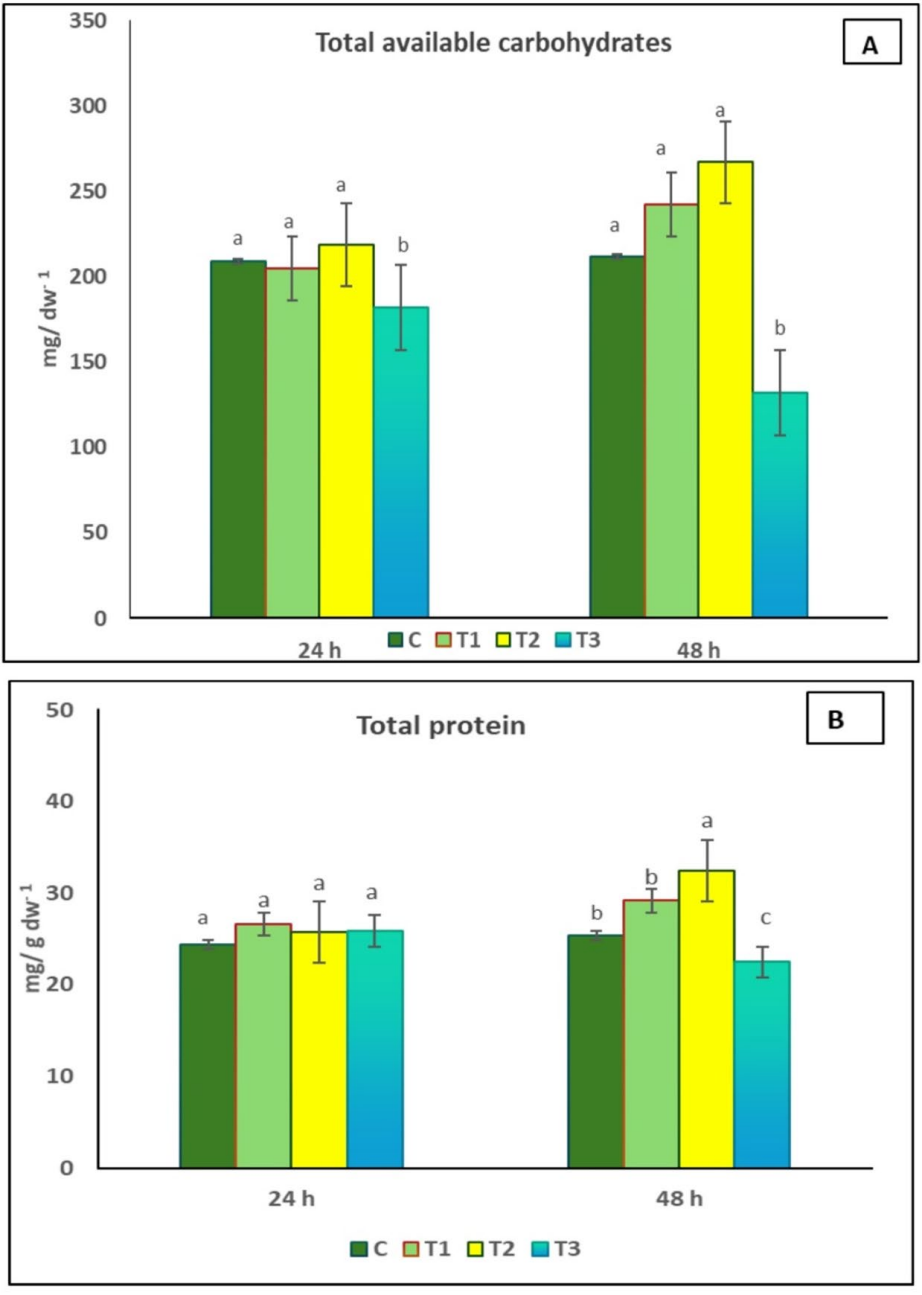
Effect of seed priming in D. regia pollen aqueous extract on total carbohydrate (A) and total protein (B) of *C. sativum*. C = no pollen treatment; T1, T2 and T3 = seed priming in 0.5, 1 and 1.5% pollen solution, respectively. The values reported in the figure are means ± standard deviation. Different letters on the bars are significantly different as evaluated by LSD Test for each pollen extract time (24 and 48 h) separately.

The effect of pollen treatments on total carbohydrate and protein contents of *C. sativum* is illustrated in Fig. 3. The results showed no significant difference in carbohydrate content between the control and the plant treated with *D. regia* pollen aqueous extract, except for T3 (1.5% pollen solution). Regarding protein content, there is no significant difference in protein content between the control plants and those treated with pollen extract for 24 h. However, treatment with 1% pollen solution for 48 h resulted in 28% increase in protein content compared to control.

### Effect of seed priming in *Delonix regia* pollen aqueous extract on total phenolic and total flavonoid contents of *Coriandrum sativum*

**Fig. 4:**
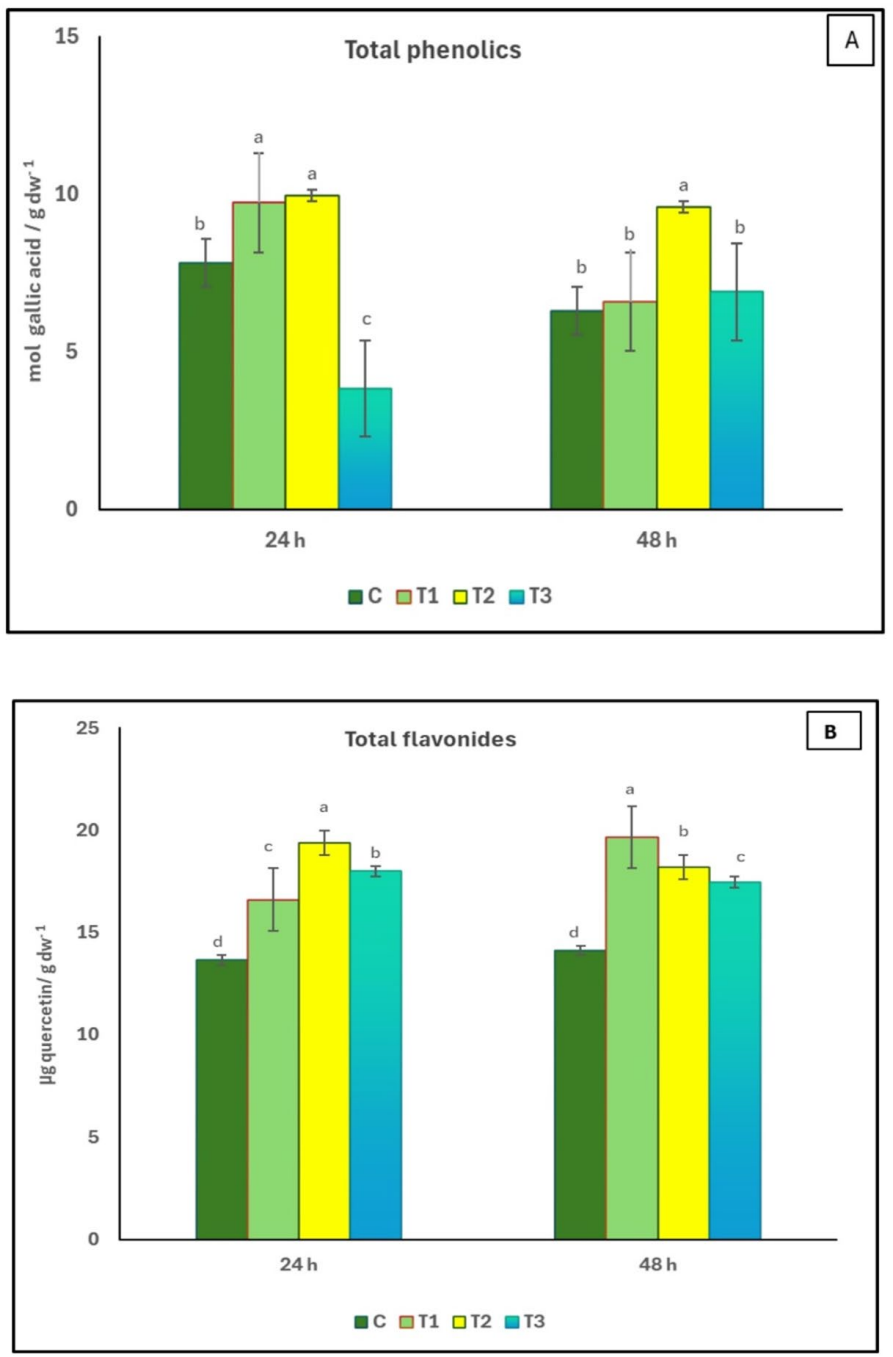
Effect of seed priming in *D. regia* pollen aqueous extract on total phenolics **(A)** and total flavonoids **(B)** of *C*. sa*tivum*. C = no pollen treatment; T1, T2 and T3 = seed priming in 0.5, 1 and 1.5% pollen solution, respectively. Different letters indicated significant difference at *p* < 0.05. The values reported in the figure are means ± standard deviation. Different letters on the bars are significantly different as evaluated by LSD Test for each pollen extract time (24 and 48 h) separately.

The effect of pollen treatments on total phenolic and total flavonoid contents of *C. sativum* is shown in Fig. 4. Compared to control, plants treated with 1% of *D. regia* pollen aqueous extract for 48 h showed significant increase in the total phenolic contents by 52.3%, whereas the total flavonoid contents increased by 39% in T1 treatment (0.5% pollen solution for 48 h) in comparison to control.

### Effect of seed priming in *Delonix regia* pollen aqueous extract on total terpene contents of *Coriandrum sativum*

**Fig. 5:**
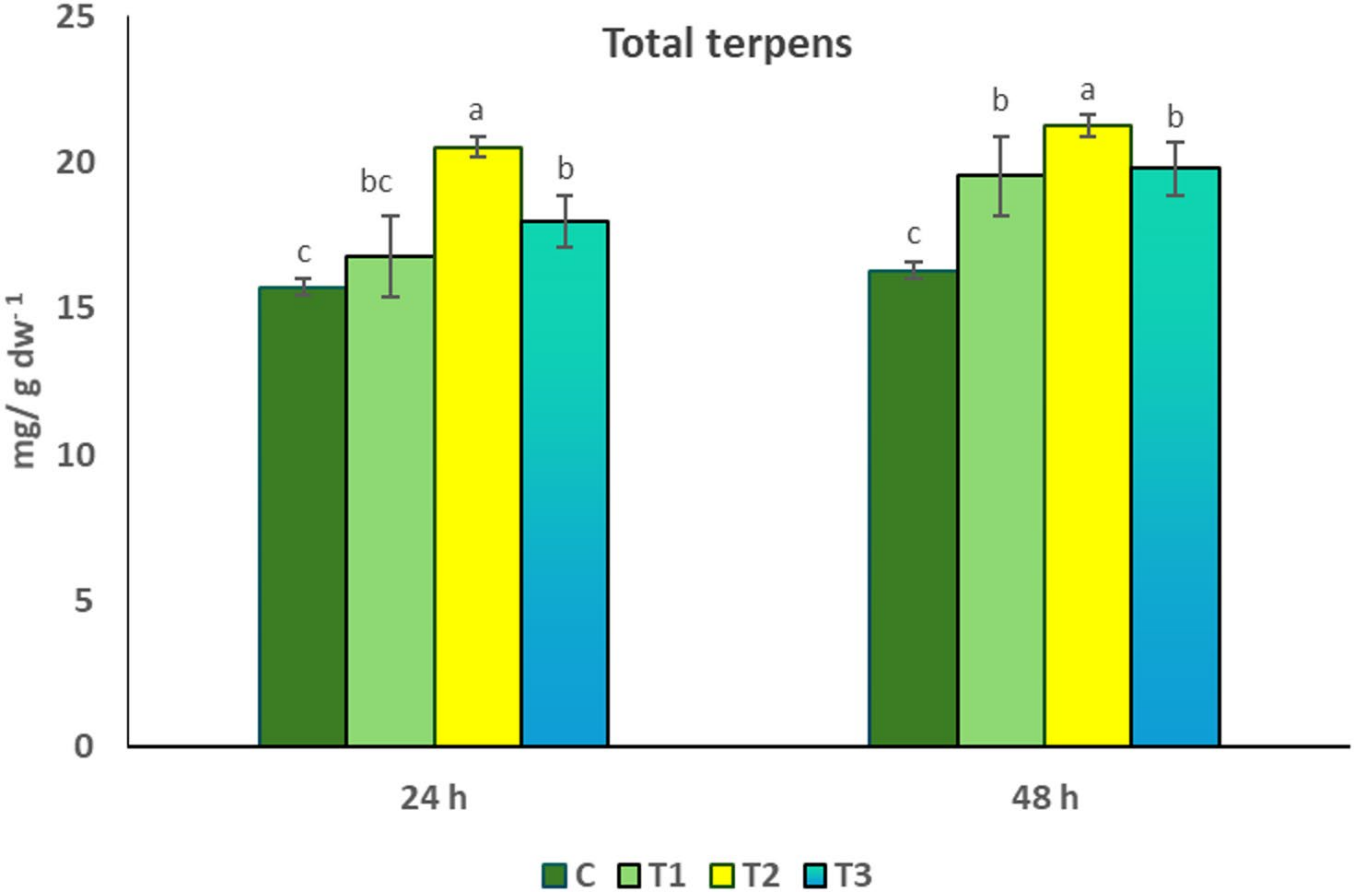
Effect of seed priming in *D. regia* pollen aqueous extract on total terpenes content of *C. sativum*. C = no pollen treatment; T1, T2 and T3 = seed priming in 0.5, 1 and 1.5% pollen solution, respectively. The values reported in the figure are means ± standard deviation. Different letters on the bars are significantly different as evaluated by LSD Test for each pollen extract time (24 and 48 h) separately.

The data presented in Fig. 5 showed that pollen treatment resulted in a significant increase in total terpene contents. The maximum value of terpenes (21.25 ± 0.297 mg/g dw^− 1^) was recorded in T2 treatment (1% of pollen extract), whereas the minimum one (15.73 ± 0.063 mg/g dm^− 1^) was observed in control plants.

### Gene expression analysis of flavonoids and terpenoids key genes in *Coriandrum sativum* treated with *Delonix regia* pollen extract

**Fig. 6:**
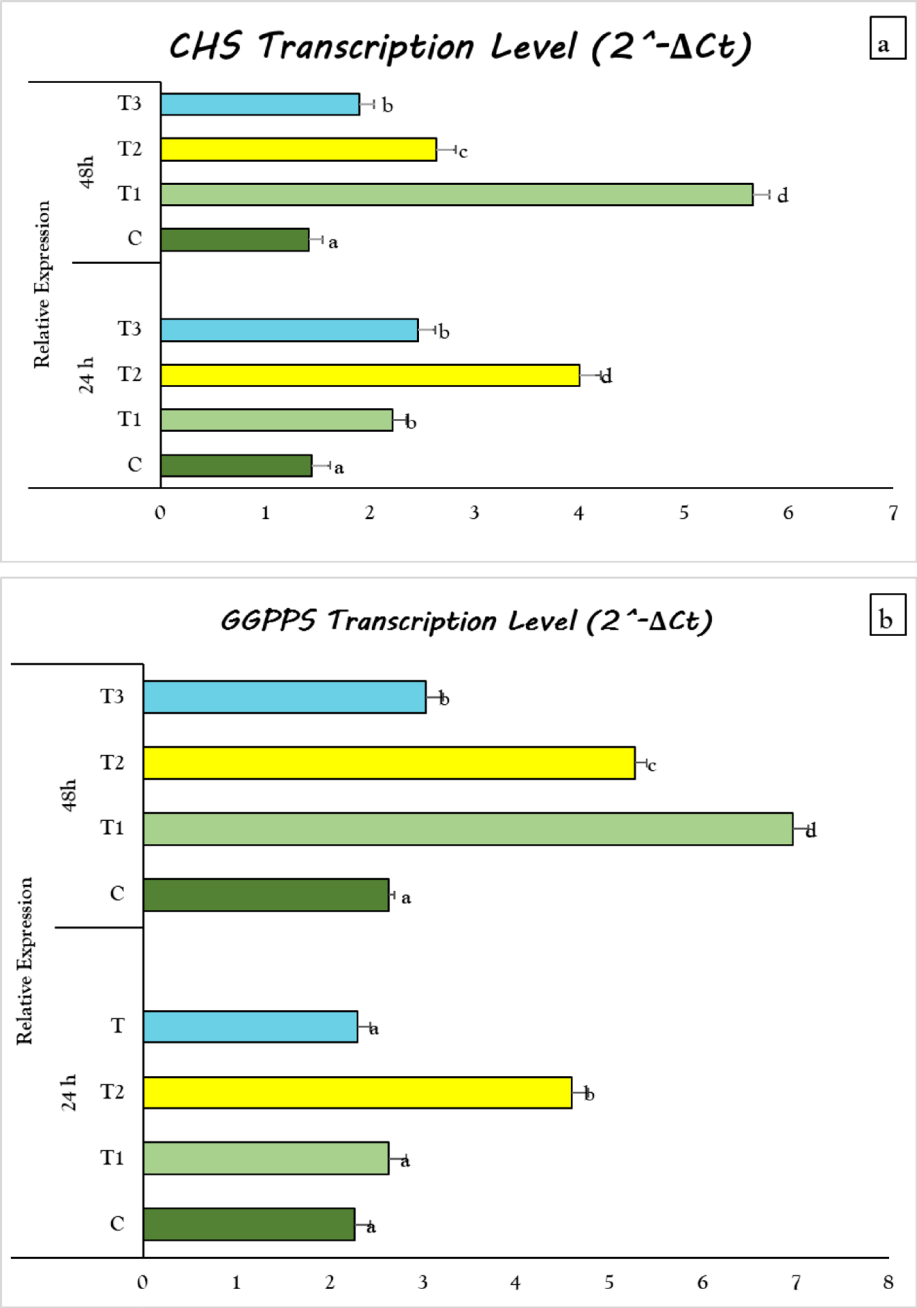
Effect of seed priming in *D. regia* pollen aqueous extract on transcription levels of CHS (**a**) and GGPPS (**b**) in of *C. sativum*. C = no pollen treatment; T1, T2 and T3 = seed priming in 0.5, 1 and 1.5% pollen solution, respectively. The values reported in the figure are means ± standard deviation. Different letters on the bars are significantly different as evaluated by Duncan’s New Multiple Range Test for each pollen extract time (24 and 48 h) separately.

This study examined the expression of chalcone synthase and geranylgeranyl diphosphate synthase, two genes implicated in the biosynthesis of flavonoids and terpenoids in *C. sativum* leaves. Chalcone synthase is the initial enzyme in flavonoid biosynthesis. The transcription levels of CHS were significantly elevated in all plants treated with pollen extract for 24 and 48 h compared to the control group. The transcript level of Chalcone synthase (CHS) increased by 2.7 and 1.4-fold in *C. sativum* treated with 1% pollen extract for 24 and 48 h, respectively, relative to control plants (Fig. 6a). Geranylgeranyl diphosphate synthase (GGPPS) initiates the formation of geranylgeranyl diphosphate, which serves as a precursor for terpenoid biosynthesis. The transcription levels of GGPPS significantly increased in all *C. sativum* treated with pollen extract for 48 h compared to control. Regarding 24 h treatment with pollen extract, GGPPS significantly increased in 1% treatment with pollen extract with an increase of 2.1-fold change (Fig. 6b).

### Data analysis

**Fig. 7:**
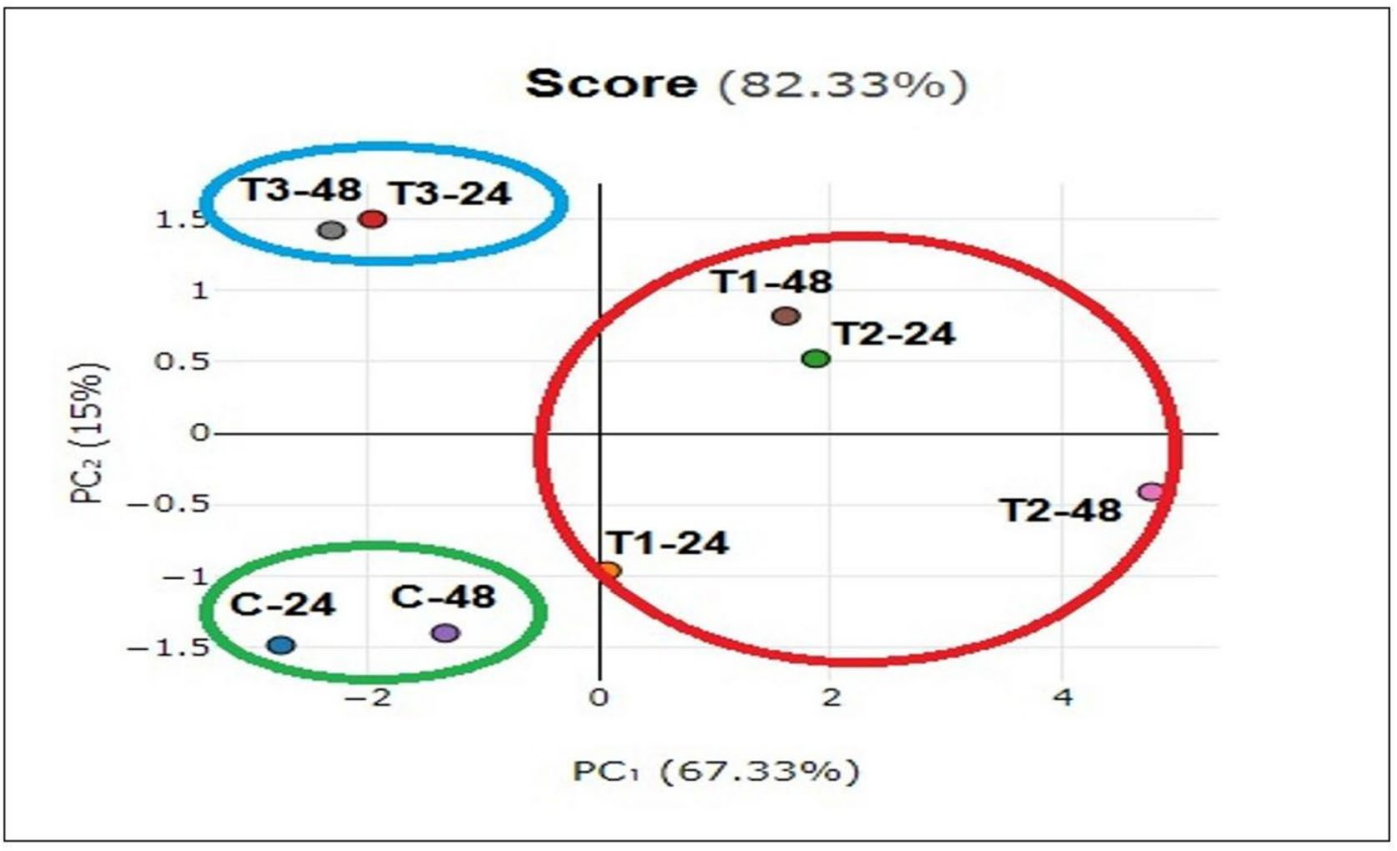
Biplot for the principal component analysis (PCA) for physiological, biochemical and molecular attributes of *C. sativum* in response to pollen treatments. C = no pollen treatment; T1, T2 and T3 = seed priming in 0.5, 1 and 1.5% pollen solution, respectively.

**Fig. 8:**
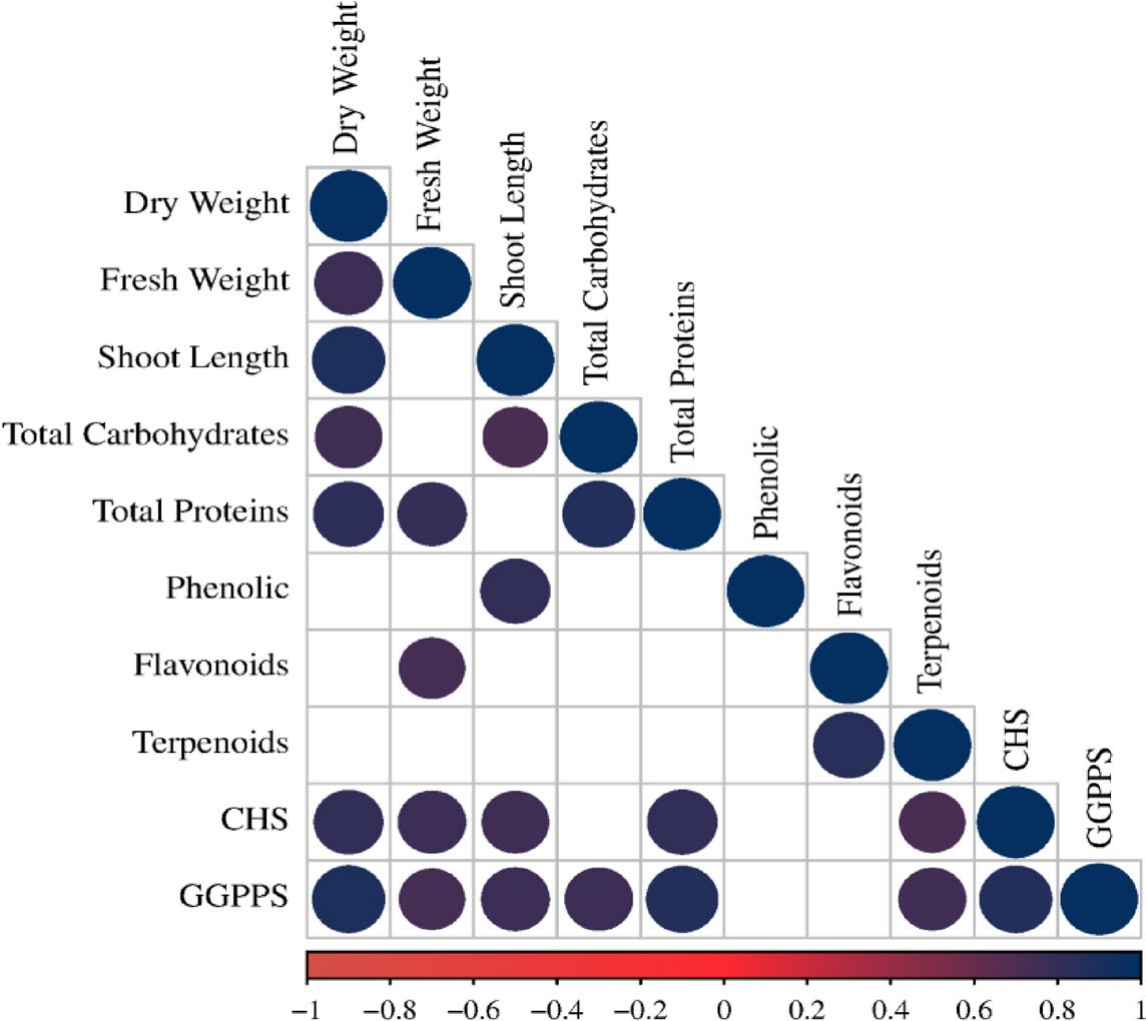
Correlogram based on the correlation coefficients between the physiological, biochemical and molecular attributes of *C. sativum* in response to pollen treatments. CHS = Chalcone synthase, GGPS = geranylgeranyl pyrophosphate synthase.

The PCA correlation between growth parameters, biochemical and gene expression of *Coriandrum sativum* as affected by pollen treatments is presented in Fig. 7. The first two components, PCA1 and PCA2, represented 67.33% and 15% of the total variation, respectively and explained the cumulative variance (82.33%) of the total variation. The PCA1 results showed a well-defined cluster consisting of pollen treatments; T1 (seed priming in 0.5% pollen solution) and T2 (seed priming in 1% pollen solution). On the other hand, PCA2 consists of two clusters; the first one includes T3 (seed priming in 1.5% pollen solution), and the second one contains the control. Correlogram based on the correlation coefficients between the physiological, biochemical and molecular attributes of *C. sativum* in response to pollen treatments are shown in Fig. 8. It is apparent from the correlogram that shoot fresh weight, total protein, total terpenes, and the expression of GGPS are positively correlated.

## Discussion

In light of increasing environmental challenges and climate change, sustainable agriculture has become essential to ensure food security and protect the environment. One of the latest trends that helps achieve these goals is the use of biostimulants, which significantly boost agricultural productivity and reduce reliance on chemical inputs^5,57^. The phytochemical analysis of pollen grains from different plant species revealed the presence of several compounds such as alkaloids, phenolic compounds, flavonoids, saponins, steroids, tannins, terpenes, proteins, amino acids, and fatty acids^58,59^. Therefore, the use of plant pollen as a biostimulant in sustainable agriculture has become increasingly popular due to its abundance of such bioactive compounds that can enhance plant growth and development.

The current study showed that priming of *C. sativum* seeds with *D. regia* pollen aqueous extract (1%) had a significant impact on shoot fresh weight, shoot dry weight and shoot length. Our findings, which demonstrate enhanced growth and biochemical content in coriander following pollen extract application, align with the growing body of evidence supporting the efficacy of biostimulants in medicinal plants. For instance, a recent study on summer savory (*Satureja hortensis*) reported that commercial biostimulants (Kadostim and Humiforte) significantly improved various physiological and biochemical parameters, underscoring the potential of these treatments to boost plant performance and valuable compound synthesis^60^. While our study utilizes a novel, natural pollen extract rather than commercial products, the consistent theme is that diverse biostimulant sources can positively modulate plant metabolism. This reinforces the strategy of exploring underutilized natural resources, such as *Delonix regia* pollen, for sustainable agricultural applications.

The findings of the current research appear consistent with another study by EL-Sayed and Darwish^61^, which found that treatment with pollen extracts resulted in significant improvements in plant height, leaf area, fruit yield, and fruit nutritional value of tomato plants under heat stress. In this context, Abo AL-Mikh^62^ reported that spraying pomegranate plants with palm pollen extract significantly improved plant height, leaf area, number of leaves, and biomass of both vegetative and root systems, as well as optimizing NPK levels.

The possible explanation of this finding is that pollen grains contain a diverse range of bioactive compounds such as vitamins, amino acids, carbohydrates, enzymes and phytohormones^3^. Phytohormones regulate a wide range of plant growth and developmental processes. They act as signaling molecules, coordinating everything from cell division and elongation to flowering and fruit ripening^63^. In addition, pollen is a rich source of minerals such as B, Zn, Se, iron Fe, Mo, Cu, Mn, Ca, Mg, K, and P^64,65^ which could serve as readily available nutrients for plants.

The present study revealed that pollen grains of *D. regia* plant contain phenolic compounds and flavonoids (Table 2), which could play a role in enhancing the growth of *C. sativum* plants. This is consistent with what Solomon et al.^19^ reported, stating that the flavonoids found in *D. regia* have significant antioxidant and anti-inflammatory effects by eliminating free radicals and regulating inflammatory pathways. In line with this, Shah et al.^66^ demonstrated the role of flavonoids in the activation of metabolic processes within canola and soybean seeds, which in turn leads to improved germination. Moreover, as signaling molecules, flavonoids modulate the expression of genes involved in plant growth and stress tolerance^67^. Priming with flavonoids can also play a role in the enhancement of the colonization of the beneficial bacteria in the rhizosphere, which in turn promote root and shoot growth^68^.

These improvements in growth parameters of the current study led to a significant enhancement in protein, phenolic, flavonoid and terpene contents and promotion of the overall physiological state of pollen-treated *C. sativum* plants. The current study revealed that the total level of terpenes was significantly higher in pollen-treated *C. sativum* plants compared to control plants. Comparable results were notified by Taha et al.^10^ who recorded that the application of pollen extracts was found to improve growth, water utilization, and antioxidant defenses in *Ocimum basilicum*, leading to increased essential oil production.

The significant increase in the total flavonoids and terpenes contents in *C*. *sativum* in response to pollen treatment may be linked to the up regulation in the transcription level of Chalconee synthase (CHS) and geranylgeranyl pyrophosphate synthase (GGPS), respectively.

This finding is concurrent with previous research that suggested chemical elicitors and biostimulants can enhance the biosynthesis pathways of secondary metabolites in plant cells through overexpression of important genes^69^. Wang et al.^70^ demonstrated that H_2_O_2_ treatment also dramatically increased the expression of the CHS gene in two hydroponic lettuce genotypes by 1.67 times. Another recent work, ABA-primed sweet sorghum seeds displayed upregulation of ten differentially expressed genes linked to flavonoid biosynthesis, including CHS^71^. Regarding biotic elicitors, Elsherif et al.^72^ demonstrated that dried and ground date palm seeds can be used as a natural elicitor to produce secondary metabolites in *Lotus arabicus.* Similarly, Alkuwayti et al.^73^, who found that the application of *Aloe vera* extract as foliar spray increased the amount of silybin in *Silybum marianum* by activating the expression of the chalcone synthase gene. Concerning the interactions between biotic and abiotic elicitors, Ji et al.^74^ employed that the application of growth-promoting bacteria *Bacillus pumilus* G5 in conjunction with silicon resulted in up-regulation of important genes involved in flavonoid biosynthesis, including CHS in *Glycyrrhiza uralensis*. Furthermore, Amani et al.^75^ stated that application of methyl jasmonate (MeJA) in combination with the culture filtrate of the fungus *Piriformospora indica* on *Ficus carica* induced the expression CHS gene.

Flavonoids present in *D. regia* pollen of the current study may play a role in enhancing the terpen ontent of *C. sativum*. Based on literature, there are few potentially mechanisms by which flavonoids and other elicitors in pollen extract trigger gene expression for terpene biosynthesis: flavonoids and other elicitors in pollen extract can trigger the expression of genes responsible for terpene biosynthesis in treated plants by activating complex signaling^76^, modulating transcription factors^77^, and integrating hormonal and stress responses^78^. These processes result in transcriptional reprogramming and upregulation of key biosynthetic genes, often involving crosstalk between flavonoid and terpene pathways.

The results of Principal Component Analysis (PCA), Fig. 7, revealed a clear distinction between *C. sativum* control plants and those treated with *D. regia* pollen extract in term of physiological, biochemical, and molecular attributes. This suggests that all pollen treatments had a noticeable impact on enhancing the growth of *C. sativum.* Notably, T2 treatment (seed priming in 1% pollen solution for 48 h) exhibiting the most distinctive biostimulant effect compared to control plants. Moreover, correlation analysis indicated that shoot fresh weight, total protein, total terpenes, and the expression of GGPS are positively correlated. GGPPS is a key structural enzyme in the terpene biosynthesis pathway, catalyzing the formation of geranylgeranyl pyrophosphate (GGPP), a precursor for various classes of terpenes^79^, thus increased expression of GGPPS genes is associated with elevated levels of specific terpenes^80^. In contrast, the downregulation of GGPPS and related genes leads to decreased terpene content^81,82^.

## Conclusion

**Fig. 9:**
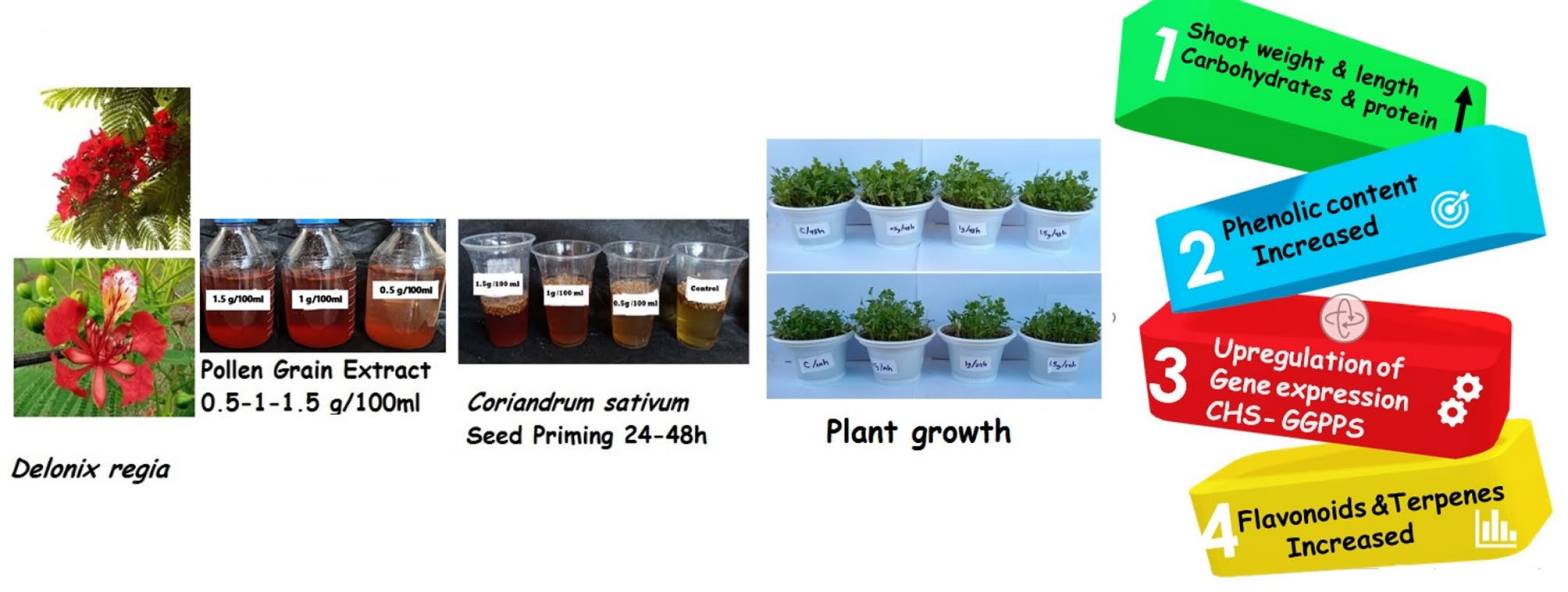
Summary of the effect of *Delonix regia* pollen extract on *Coriandrum sativum* plants.

In conclusion, our study demonstrates that seed priming with *Delonix regia* pollen aqueous extract, particularly at 1% for 48 h, acts as an effective biostimulant for *Coriandrum sativum*. This treatment significantly enhances growth parameters and boosts the accumulation of valuable phytochemicals, including phenolics, flavonoids, and terpenes. The observed upregulation of CHS and GGPS genes provides a molecular basis for the increased production of these secondary metabolites. This work establishes *D. regia* pollen as a promising, natural, and sustainable tool for improving coriander quality and yield. Future research should focus on isolating the specific bioactive compounds within the pollen responsible for this elicitation effect and validating these results under field conditions (Fig. 9).

## Data Availability

All data generated or analyzed during this study are included in this published article.
